# Mutation spectrum of *PTS* gene in patients with tetrahydrobiopterin deficiency from jiangxi province

**DOI:** 10.3389/fgene.2022.1077729

**Published:** 2022-12-13

**Authors:** Kang Xie, Baitao Zeng, Liuyang Zhang, Shaohong Chen, Yongyi Zou, Huizhen Yuan, Shuhui Huang, Feng Wang, Qing Lu, Yanqiu Liu, Bicheng Yang

**Affiliations:** Jiangxi Provincial Key Laboratory of Birth Defect for Prevention and Control, Jiangxi Maternal and Child Health Hospital, Nanchang, China

**Keywords:** hyperphenylalaninemia (HPA), tetrahydrobiopterin deficiency, 6-pyruvoyltetrahydropterin synthase gene, mutation spectrum, jiangxi province

## Abstract

**Background:** Hyperphenylalaninemia (HPA) is the most common inborn error in amino acid metabolism. It can be primarily classified into phenylalanine hydroxylase (PAH) deficiency and tetrahydrobiopterin (BH4) deficiency. BH4 deficiency (BH4D) is caused by genetic defects in enzymes involved in the biosynthesis and regeneration of BH4. 6-pyruvoyl-tetrahydropterin synthase (PTPS/PTS), which is encoded by the *PTS* gene, participates in the biosynthesis of BH4. PTPS deficiency (PTPSD) is the major cause of BH4D. In this study, we investigated that the prevalence of BH4D in Jiangxi province was approximately 12.5 per 1,000,000 live births (69/5,541,627). Furthermore, the frequency of BH4D was estimated to be 28.8% (69/240) in the HPA population of Jiangxi. In this study, we aimed to characterize the mutational spectrum of the *PTS* gene in patients with PTPSD from Jiangxi province.

**Method:** Newborn screening data of Jiangxi province from 1997 to 2021 were analyzed and 53 families with PTPSD were enrolled for the analysis of the *PTS* gene variants by Sanger sequencing.

**Results:** 106 variants were identified in 106 alleles of 53 patients with PTPSD, including 13 types of variants reported previously, and two novel variants (c.164-36A>G and c.146_147insTG). The predominant variant was c.259C>T (47.2%), followed by c.84-291A>G (19.8%), c.155A>G (8.5%), c.286G>A (6.6%) and c.379C>T (4.7%).

**Conclusion:** The results of this study can not only provide guidance for the molecular diagnosis and genetic counseling in cases of PTPS deficiency but also enrich the *PTS* mutation database.

## Introduction

Tetrahydrobiopterin deficiency (BH4D) can result in functional disturbances in the central nervous system (Souza et al., 2018). It is caused by genetic defects in enzymes involved in the biosynthesis and regeneration of tetrahydrobiopterin (BH4) (Himmelreich et al., 2021). In China, the average incidence of BH4D is approximately 3.8 per 1,000,000 live births (Yuan et al., 2021). The *de novo* synthesis of BH4 from GTP requires the participation of three enzymes including GTP cyclohydrolase I, 6-pyruvoyltetrahydropterin synthase (PTPS/PTS) and sepiapterin reductase (Tendi et al., 2022). In addition, dihydropteridine reductase (DHPR) can contribute to the production of BH4 *via* an alternative or salvage pathway (Himmelreich et al., 2021). PTPS deficiency (PTPSD) caused by mutations in the *PTS* gene, is the most common disorder in BH4D, followed by DHPR deficiency (DHPRD) caused by defects in the quinoid dihydropteridine reductase (*QDPR*) gene (Opladen et al., 2012).

As a cofactor of the phenylalanine hydroxylase (PAH) enzyme that catalyzes the transfer of L-tyrosine (Tyr) from L-phenylalanine (Phe), BH4D causes a build-up of Phe in the blood, which leads to hyperphenylalaninemia (HPA) (Tendi et al., 2022). Approximately 98% of HPA cases are related to PAH deficiency (PAHD) and only 2% of HPA cases are caused by BH4D (Zurflüh et al., 2008; Gundorova et al., 2021). Although PAHD and BH4D are both identified as HPA, the treatment methods used are different (Singh et al., 2014). The analysis of urinary pterins profile, and BH4 loading test results, and the determination of DHPR activity in red blood cells can be used in the differential diagnosis between PAHD and BH4D (Opladen et al., 2011; Elhawary et al., 2022). BH4D gradually leads to intellectual disability and age-dependent movement disorders, which can be prevented with the early initiation of effective treatment (Opladen et al., 2012, 2020). Additionally, the early confirmation of diagnosis is critical for the dietary treatment and drug treatment of BH4D (Campistol Plana, 2019; Opladen et al., 2020). Newborn screening (NBS) is cost-beneficial and can save considerable public expenditure (LEE et al., 2014). Many countries have also made great advances in the early diagnosis and treatment of BH4D using NBS (Ye et al., 2002; Niu, 2011; Li et al., 2018; Souza et al., 2018). There are considerable differences geographical location and ethnic composition in the various provincial regions of China (Li et al., 2019). BH4D prevalence was higher in the northern regions (4.1 per 1,000,000) of China than in the southern regions (1.6 per 1,000,000). Jiangxi province has the highest rate of BH4D prevalence among the provinces of China (Yuan et al., 2021). In addition, the proportion of PTPSD-related variants in mainland Chinese patients with BH4D may be as high as 97.73% (Li et al., 2018; Ye et al., 2018). Thus, it is essential to construct the *PTS* mutation spectrum at the provincial level, which will lay the foundation for accurate diagnosis and individualized genetic counseling.

In this study, we analyzed the newborn screening data of Jiangxi province from 1997 to 2021. 69 newborns were diagnosed with BH4D in all. Following this, we recruited 53 patients with PTPSD and extracted DNA samples from the patients and their parents to identify mutations in the *PTS* gene using Sanger sequencing. Based on the results, we summarized the *PTS* mutation spectrum in Jiangxi province for the first time.

## Materials and methods

### Patients

PTPSD patients came from the positive cases encountered in newborn screening form October 1997 to December 2021. The study recruited 53 patients with PTPSD, 29 males and 24 females. All participants originated from Jiangxi province. Patients were diagnosed with HPA when the ratio of blood Phe/Tyr concentration exceeded two and the blood Phe concentration exceeded 120 µmol/L. The analysis of urinary pterins profile and BH4 loading test results and the measurement of DHPR activity in red blood cells were used to diagnose BH4D (abnormity of pterins profile and normality of DHPR). Patients with BH4D were diagnosed with PTPSD with the increase in neopterin levels and decrease in biopterin levels, according to the consensus about the diagnosis and treatment of hyperphenylalaninemia in China (Yang et al., 2014). Shanghai Xinhua Hospital completed the analysis of urinary pterins profile and the measurement of DHPR activity in red blood cells. The signing of the informed consent was carried out in parents or legal guardians of all study participants. The research obtained approval from the Clinical Research Ethics Committees of Jiangxi maternal and child health hospital, Nanchang, China.

### Genotype analysis

Taking advantage of a QIAamp DNA Mini Kit (Qiagen), genomic DNA was extracted only from the whole blood in patients diagnosed with PTPSD and their parents. The causative variant of *PTS* gene was identified in each family using PCR and Sanger sequencing. To amplify all the coding exons and primary splice sites, six pairs of primers were designed by Primer-BLAST (https://www.ncbi.nlm.nih.gov/tools/primer-blast/) (Supplementary Table S1) and synthesized by a company (Tsingke, ChangSha). According to the manufacturer’s protocol, the PCR amplification reaction system was configured with a 2x Taq PCR Master MixII (KT211,TIANGEN). The PCR program was 95°C for 5 min for initial denaturing, then 30 cycles at 95°C for 40 s, at 57°C for 30 s, and at 72°C for 35 s, followed by a final extension of 8 min at 72°C in a T100 Thermal Cycler for the Classroom (biorad). Six of the PCR products were sent to a sequencing provider (Tsingke, ChangSha) for Sanger sequencing. Mutation analysis was performed by sequence alignment with the PTS transcript (NM_000317). Information on the Mutation naming scheme can be found at the HGVS site (https://www.HGVS.org/varnomen). The definite causative variants were elected by going through the Human Gene Mutation Database (HGMD, http://www.hgmd.cf.ac.uk/ac/validate.php) and International Database of Patients and Mutations causing BH4-responsive HPA/PKU (BIOPKU, http://www.biopku.org/home/home.asp) from among variants detecting by sequencing.

### Statistical analysis

Graph- Pad Prism version 8.0.1 software was adopted to study and analyze statistically. Measurement data were compared with analysis of variance. Calculators’ information was displayed as a percentage.

## Results

### Mutation analysis

A total of 5,541,627 newborns were screened for neonatal diseases at our center between 1997 and 2021, and HPA was detected in 240 patients. In the past 25 years, 69 HPA cases have been diagnosed as BH4D patients in total. These included 68 cases with PTPSD and 1 case with DHPRD. The frequency of BH4D was estimated to be 28.8% (69/240) in the HPA population of Jiangxi province, which was considerably higher than the 3.9% observed in the Chinese population on average (Yuan et al., 2021). Therefore, we speculated that the prevalence of BH4D in Jiangxi province was approximately 12.5 per 1,000,000 live births (69/5,541,627). Among 68 cases of PTPSD, 53 cases were recalled for further genetic diagnosis, whereas the 15 remaining cases were lost to follow-up.

**FIGURE 1:**
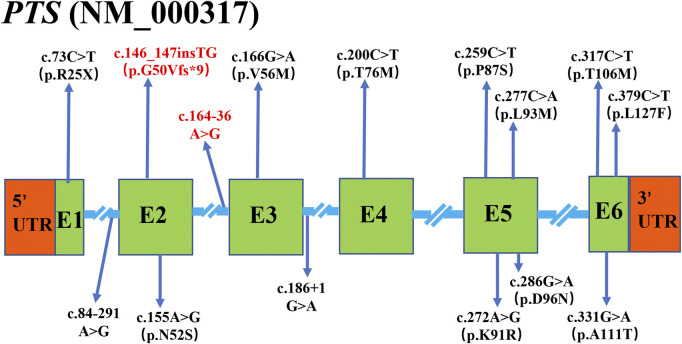
The results of Sanger sequencing in 53 patients with PTPSD. 15 kinds of variants located in all the coding exons (E) and primary splice sites (blue line) of PTS gene. The novel mutation was marked with the red color.

The results of Sanger sequencing showed that 15 kinds of variants were found overall in 106 alleles of *PTS* gene (Figure 1). They were in all over the six coding exons, whereas splicing variants were only present in intron 1 and 2 (Figure 1). Furthermore, pedigree analysis of the probands family was performed to evaluate the cis or trans phase of the variants. All disease-causing mutations were inherited from his or her parents. In 53 families, PTPSD was caused by the compound heterozygous mutation or homozygous mutation of *PTS*.

15 kinds of variants located in all the coding exons (E) and primary splice sites (blue line) of *PTS* gene. The novel mutation was marked with the red color.

### Genotyping analysis

**FIGURE 2:**
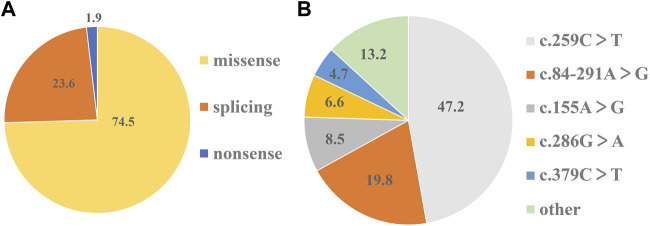
Shares of mutation types and prevalent frequencies of detected variants. **(A)**The percentage of three types of variants in 106 detected variants respectively. **(B)** The percentage of each variant in 106 detected variants respectively.

On the basis of mutation types, the detected variants were divided into three groups. The missense variants accounted for 74.5%, followed by splicing variants (23.6%) (Figure 2A). As a result, 10 missense variants existed in most of the patients: c.155A>G (N52S), c.166G>A (V56M), c.200C>T (T67M), c.259C>T (P87S), c.272A>G (K91R), c.277C>A (L93M), c.286G>A (D96N), c.317C>T (T106M), c.331G>A (A111T), and c.379C>T (L127F) (Table 1). Three splicing variants appeared in 21 patients with PTPSD: c.84-291A>G (IVS1-291A>G), c.164-36A>G (IVS2-36 A>G), and c.186 + 1G>A (IVS3 + 1G>A) (Table 1). In particular, a non-sense variant and a frameshift variant were detected in this paper: c.73C>T (R25X) and c.146_147insTG (G50Vfs*9) (Table 1). The prevalent frequencies of variants from high to low was as follows: c.259C>T (50/106, 47.2%), c.84-291A>G (21/106, 19.8%), c.155A>G (9/106, 8.5%), c.286G>A (6/92, 6.6%), and c.379C>T (5/92, 4.7%) (Figure 2B). The remaining 13.2% consisted of some extremely low frequency variants (Figure 2B).

**TABLE 1:** Genotyping analysis of 53 patients with PTPSD in Jiangxi province.

Location	Nucleotide change	Amino acid change	Character of mutation	No. of alleles	Allele frequency (%)
exon 1	c.73 C>T	R25X	Non-sense	1	0.94
intron1	c.84-291 A>G	IVS1-291 A>G	Splice	21	19.81
exon 2	c.146_147insTG	G50Vfs*9	Frameshift	1	0.94
exon 2	c.155 A>G	N52S	Missense	9	8.49
intron2	c.164-36 A>G	IVS2-36 A>G	Splice	1	0.94
exon 3	c.166 G>A	V56M	Missense	1	0.94
intron3	c.186 + 1 G>A	IVS3+1G>A	Splice	3	2.83
exon 4	c.200 C>T	T67M	Missense	2	1.89
exon 5	c.259 C>T	P87S	Missense	50	47.17
	c.272 A>G	K91R	Missense	1	0.94
	c.277 C>A	L93M	Missense	1	0.94
	c.286 G>A	D96N	Missense	7	6.60
exon 6	c.317 C>T	T106M	Missense	1	0.94
	c.331 G>A	A111T	Missense	2	1.89
	c.379 C>T	L127F	Missense	5	4.72
identified				106	100.00
Not identified				0	0.00
Total number of alleles				106	100.00

### Two novel mutation of *PTS*

**FIGURE 3:**
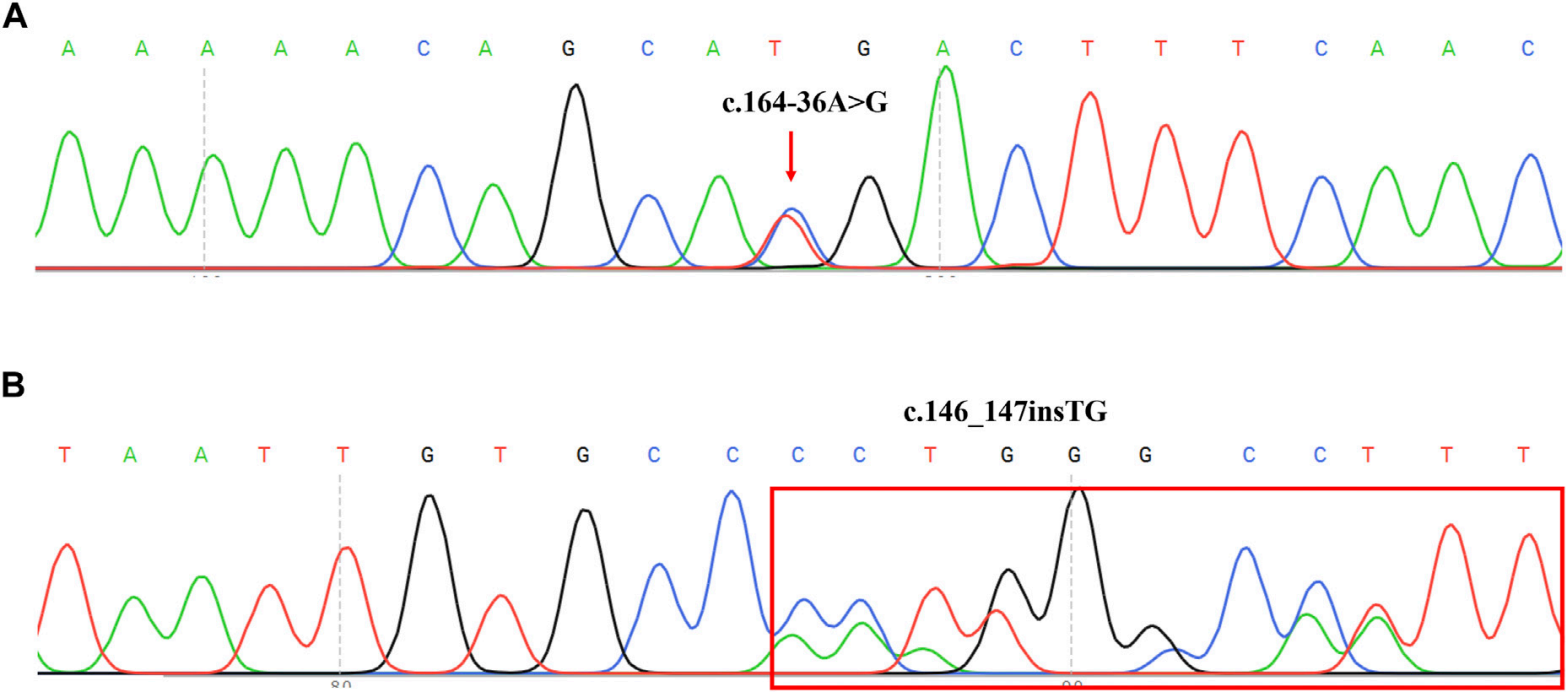
Sequencing results of two novel mutation on *PTS* reverse sequences in two PTPSD probands. **(A)** Sequence chromatogram indicated a A-to-G transition of nucleotide 164-36. The transcript of *PTS* gene (NM_000317) was chosen. **(B)** Sequence chromatogram indicated insertion of TG between nucleotide 146 and 147. The transcript of *PTS* gene (NM_000317) was chosen.

DNA sequence assays showed that a proband had two splicing variants located in intron1 and intron2 (c.164-36A>G and c.84-291A>G) respectively. Further, the sources of two variants were examined in her parents respectively. In addition, the sequencing result of another proband revealed c.146_147insTG was in compound heterozygosity with c.379C>T. Previous research had found c.84-291A>G and c.379C>T were the common variants in the Chinese PTPSD patients (Ye et al., 2013). However, c.164-36A>G and c.146_147insTG (Figure 3) had never been reported in previous research and not recorded in HGMD and BIOPKU. The two novel mutations were not present in the population frequency databases.

## Discussion

In BH4D, the activity of PAH, tyrosine hydroxylase and tryptophan hydroxylase are reduced, which hampers the synthesis of neurotransmitters in the brain (Li et al., 2022). Most children who develop untreated BH4D exhibit severe neurological symptoms after 3 months of life (Wang et al., 2006; Manti et al., 2020). NBS for HPA and proper metabolic management can aid the early diagnosis and treatment of BH4D, which can significantly improve the prognosis (Wang et al., 2006; Vatanavicharn et al., 2009; Shintaku and Ohwada, 2013). As an effective and cost-beneficial strategy for BH4D, many countries have conducted NBS in such cases. (Li et al., 2018; Souza et al., 2018; Ray et al., 2022).

Only a few studies have been conducted on BH4D prevalence in live births. (Tada et al., 1984; Niu et al., 2010; Yuan et al., 2021). The global BH4D prevalence is 1 per 1,000,000 live births and differs worldwide (Blau et al., 1996). We speculated that the prevalence of BH4D in Jiangxi province was approximately 12.5 per 1,000,000 live births from 1997 to 2021, whereas that in Minas Gerais of Brazil was 2.1 per 1,000,000 live births from 1993 to 2012 (Souza et al., 2018). Moreover, the average prevalence of BH4D in China was 3.8 per 1,000,000 live births (Yuan et al., 2021). The highest prevalence was observed in eastern China (5.9 per 1,000,000), and Jiangxi province had the highest rate among the provinces of China (10.6 per 1,000,000) (Yuan et al., 2021). While the prevalence we obtained is similar to the ones reported earlier, our data are completer and more accurate of local situation. Furthermore, we observed that the proportion of BH4D among HPA cases was 28.8% in Jiangxi province, which was much higher than the prevalence in Italy (10%) (Blau et al., 1996), Turkey (15%) (Blau et al., 1996) and Iran (12.3%) (Khatami et al., 2017), and even higher than that in Mexican (9.8%) (Vela-Amieva et al., 2022), France (1.87%) (Dhondt and Hayte, 2002) and Russia (0.5%) (Gundorova et al., 2021). However, the incidences in Taiwan and Jordan are more than 30% (Niu, 2011; Carducci et al., 2020). China’s average frequency of BH4D in HPA cases was 3.9%, but noticeable regional differences were observed (Yuan et al., 2021). The Jiangxi province is located in south-central China. The southern region had the highest frequencies (15.1%), followed by the eastern (5.3%) and southwestern (5.1%) regions (Yuan et al., 2021). The incidence in Jiangxi province was well above those in other provinces of China, such as Shandong province (10.1%) (Han et al., 2015). In Jiangxi province, the current scenario of BH4D is different from that in other places. Therefore, constructing own spectrum might be more suitable here.

A relatively high prevalence of PTPSD was also reported in the Arab population, the most common variant being c.238A>G (33%) (Almannai et al., 2019). c.1222C>T was the most common variant in Russia (Gundorova et al., 2019), whereas in numerous studies have reported the c.259C>T was found to be the most common variant in mainland China (Liu et al., 1998; Chiu et al., 2012). In this study, the most frequent mutation was c.259C>T, which was also observed in Shandong province (Han et al., 2015). The most common variant varied significantly across regions but was similar in East Asian populations due to a founder effect (Chiu et al., 2012). In China, c.286G>A was a common variant among the northern populations, and c.155A>G was a common variant among the southern populations (Liu et al., 1998, 2001). Therefore, prioritizing the genotype analysis of the hotspot region in children with BH4 deficiency is critical to improving the diagnostic efficiency and reducing costs. The Mutation spectrum of the *PTS* gene in this region is generally consistent with that reported in previous studies (Liu et al., 1998; Chiu et al., 2012; Ye et al., 2013; Li et al., 2018), but also has a certain uniqueness. The incidence of c.84-291A>G in this region was relatively high, close to that of c.259C>T. The incidence of c.379C>T was also relatively high. The *PTS* gene mutation spectrum can serve as an important reference for the early diagnosis, treatment, genetic counseling, and prenatal diagnosis of BH4D in Jiangxi province. In addition, the identification of two novel mutations, c.164-36A>G and c.146_147insTG in this study enriched human genetic variation database.

In summary, we analyzed the newborn screening data from 1997 to 2021 in Jiangxi province. The prevalence of BH4D in Jiangxi province was approximately 12.5 per 1,000,000 live births (69/5,541,627), and the proportion of BH4D among HPA cases was 28.8% (69/240). For the first time, we successfully constructed the *PTS* mutation spectrum in Jiangxi province. This mutation spectrum is meaningful and can help guide molecular diagnosis and effective genetic counseling in PTPSD.

## Data Availability

The raw data supporting the conclusions of this article will be made available by the authors, without undue reservation.
